# Video calls from lay bystanders to dispatch centers - risk assessment of information security

**DOI:** 10.1186/1472-6963-11-244

**Published:** 2011-09-30

**Authors:** Stein R Bolle, Per Hasvold, Eva Henriksen

**Affiliations:** 1 Norwegian Centre for Integrated Care and Telemedicine, University Hospital of North Norway, N-9038 Tromsø, Norway; 2 Division of Trauma Care and Pre-Hospital Services, University Hospital of North Norway, N-9038 Tromsø, Norway

## Abstract

**Background:**

Video calls from mobile phones can improve communication during medical emergencies. Lay bystanders can be instructed and supervised by health professionals at Emergency Medical Communication Centers. Before implementation of video mobile calls in emergencies, issues of information security should be addressed.

**Methods:**

Information security was assessed for risk, based on the information security standard ISO/IEC 27005:2008. A multi-professional team used structured brainstorming to find threats to the information security aspects confidentiality, quality, integrity, and availability.

**Results:**

Twenty security threats of different risk levels were identified and analyzed. Solutions were proposed to reduce the risk level.

**Conclusions:**

Given proper implementation, we found no risks to information security that would advocate against the use of video calls between lay bystanders and Emergency Medical Communication Centers. The identified threats should be used as input to formal requirements when planning and implementing video calls from mobile phones for these call centers.

## Background

Cardiac arrest, accidents and traumas are leading causes of death worldwide [1-3]. First rescue activities performed by lay bystanders, such as calling for help, opening of airways, and cardio-pulmonary resuscitation, save lives. Emergency Medical Communication Centers (EMCCs) assist bystanders via telephone, saving time and improving care [4,5]. EMCC operators (dispatchers) often have to act on limited information, as the description given by bystanders can be lacking or misleading [4,6,7].

Videoconference enabled mobile phones can be sophisticated tools for dispatcher assisted resuscitation [7,8], and videoconferencing can improve the confidence of lay rescuers [9]. Videoconferencing used in communication between bystanders and EMCCs would enable dispatchers to see the patient and the scene of accident, and may better instruct bystanders on correct action [7,10,11]. The Federal Communications Commission (FCC) in the USA announced in November 2010 that America's 9-1-1 system should be revolutionized by harnessing the life-saving potential of text, photo, and video in emergencies [12]. Although a majority of the emergency calls come from mobile phones [12], call centers currently lack the technical capability to use the full potential of these new technologies.

In healthcare, information security and safety are vital parts of the trust between the public and the care providers. In most countries this is regulated through laws and professional standards. Possible undesired effects should be identified before the implementation of video calls in EMCCs. In this study, the security challenges of using mobile telephones for videoconferencing between lay rescuers and EMCCs were analyzed through a qualitative risk assessment of the information security aspects.

## Methods

**Figure 1:**
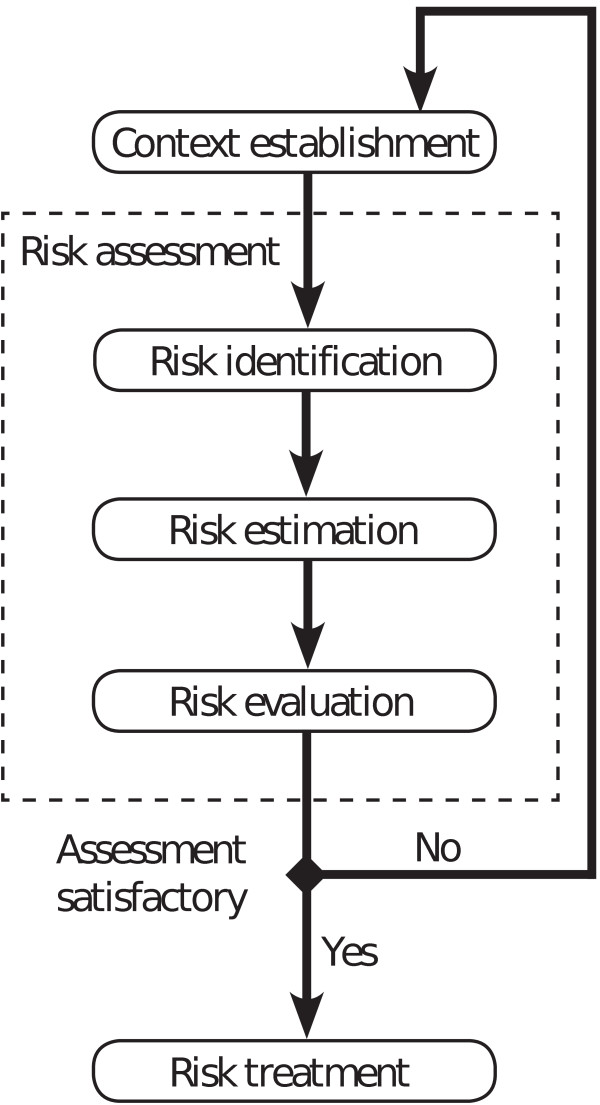
**The workflow of risk assessment according to the information security standard ISO/IEC 27005:2008**.

Risk assessment is a systematic approach for describing and calculating risks of undesired events. We conducted risk assessment of information security related to the use of videoconference calls with mobile phones between lay bystanders and EMCCs during medical emergencies. Our risk assessment was based on the information security standard ISO/IEC 27005:2008 developed by the International Organization for Standardization (ISO) and the International Electrotechnical Commission (IEC) [13]. In this standard, risk assessment is described as a process consisting of risk identification, risk estimation and risk evaluation. Risk assessment is performed after context establishment, and the process may be iterative (Figure 1).

### Risk assessment group, workflow and time frame

Risk assessment was performed by a group, led by a risk assessment expert (EH). Group participants were chosen from our own institution, based on competencies and background, such that different areas of expertise were covered. One anesthetist nurse, one anesthesiologist, one lawyer, and two computer scientists took part in this group, which started its work in 2006. After one iteration of risk assessment including three group meetings, we found that a better understanding of the intended service was needed, and agreed to postpone further iterations until the completion of a research project [7,9,14]. We expanded our group with a dispatcher nurse who had used videoconferencing in scenarios of simulated cardiac arrest, and the risk assessment was completed through another two iterations with email discussions and eight group meetings during 2009 and 2010.

### Context establishment

The context for this risk assessment was set by describing the service, legal requirements and definitions. Legal requirements for communication of sensitive patient-identifiable information is set by national and European legislation [15-17]. The consequence of risks were defined in three categories (low, medium, high), and values for likelihood were described using four categories (low, medium, high, very high) (Table 1). Risk (R) is the product of consequence (C) and likelihood (L): *R *= *C *× *L*. In qualitative risk assessment, risk is illustrated in a two dimensional matrix as a combination of consequence and likelihood. We defined three levels for risks; low, moderate and severe (Table 1). Threats with severe risk are usually unacceptable. If they cannot be avoided or their risk reduced, it may imply that the new service should not be implemented.

**Table 1:** Definitions of consequence, likelihood and risk level

Category	Description
**Consequence**	

Low	For the hospital or the service: No violation of law; or negligible economic loss which can be restored; or small reduction of reputation in the short run.
	For the patient: A minor impact on health; or negligible economic loss which can be restored; or small reduction of reputation in the short run.
Medium	For the hospital or the service: Offence, less serious violation of law which results in a warning or a reprimand; or economic loss which can be restored; or reduction of reputation that may influence trust and respect.
	For the patient: A minor temporary impact on health; or economic loss which can be restored; or small reduction of reputation caused by revealing of less serious information (e.g. blood pressure level).
High	For the hospital or the service: Violation of law which results in penalty or fine; or a large economic loss which cannot be restored; or serious loss of reputation that will influence trust and respect for a long time.
	For the patient: Death or permanent reduction of health; or a large economic loss which cannot be restored; or serious loss of reputation caused by revealing of sensitive and offending information.

**Likelihood**	

Low	Rare, occurs less than every 100th connection. Detailed knowledge about the system is needed; or special equipment is needed; or it can only be performed deliberately.
Medium	May happen, occurs between every 10th and every100th connection. Normal knowledge about the system is sufficient; or normally available equipment can be used; or it can be performed deliberately.
High	Quite often, occurs between every 3rd and every10th connection. Can be done with minor knowledge about the system; or without any additional equipment being used; or it can be performed by wrong or careless usage.
Very high	Very often, occurs more often than every 3rd connection. Can be done without any knowledge about the system; or without any additional equipment being used; or it can be performed by wrong or careless usage.

**Risk level**	

Low	Acceptable risk. The service can be used with the identified threats, but the threats must be observed to discover changes that could raise the risk level.
Moderate	Can for this service be an acceptable risk, but for each threat the development of the risk should be monitored to consider whether necessary measures have to be implemented.
Severe	Not acceptable risk. Cannot start using the service before risk reducing treatment has been implemented.

### Risk assessment

Threats to information security with consequences for the organization or patients were identified. We considered threats related to legislation and regulations, economic consequences, reputation, life, and health.

Identification of threats was performed as a structured brainstorming in the risk assessment group. All ideas for possible risks were noted and no risks were censored or rejected at this point. During risk assessment we focused on confidentiality (c), quality (q), integrity (i), and availability (a) of information, terms defined by Norwegian legislation as the aspects of information security [15,16]. Every threat was described and given a unique identifier where the first character was used to indicate the type of security aspect (c, q, i, a).

Each threat was analysed by the team for the consequence and the likelihood that it would occur, according to definitions (Table 1). Risk assessment was done for the new service relative to the existing service with audio only communication. This means that risks in the existing service were excluded, unless the new service would change the risk level.

The identified threats were placed in a two dimensional matrix according to their consequence and likelihood. Each threat was evaluated, and possible actions to reduce the risks were suggested. The process was continued until we reached group consensus.

## Results

**Figure 2:**
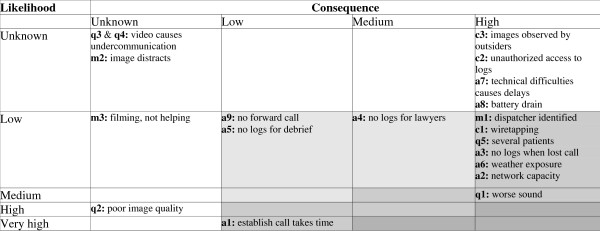
**Risk matrix presenting the identified threats with identifier and short description**. Darker shades of grey indicates higher level of risk: light grey low risk, medium grey moderate risk and darkest grey severe risk. White background is used for threats with unknown risk.

Twenty distinct threats and unwanted situations were identified and described (Table 2). The likelihood and consequence were estimated for each threat. The risk matrix presents all threats with their id, short description and risk level as a combination of likelihood and consequence (Figure 2). No threats had a severe risk level, but threats with a high level of consequence should be watched closely, as an increase in likelihood can make these threats severe. We were not able to conclude on likelihood or consequence for nine threats, either because it would be dependent on the implementation of the technology, or related to issues that can only be answered through clinical trials. It is possible that these threats could have an unacceptable severe risk.

**Table 2:** Description of threats

Threat id	Description
**Threats to quality**	

q1	Sound quality with mobile phone videoconferencing is usually worse than regular calls between mobile phones. Reasons include poor bandwidth and mobile phones usually in loudspeaker mode during video calls, often with disturbing background noise. This may result in misunderstandings, lost information and delays.
q2	Poor image quality is a common problem with video calls from mobile phones. Although likely to improve with improved technology, camera shake, poor light and weather conditions will influence on the image quality. Some image quality problems are due to current methods for video compression.
q3	The caller may believe that the image is a sufficient description, therefore not describing the situation appropriately, which leads to misinterpretations.
q4	The dispatcher may believe the image describes the situation sufficiently, and therefore do not ask for important information, which leads to misinterpretations.
q5	When there are several patients in the same accident or emergency, it is possible to mix-up images from one patient to what is said about another patient. The image may clarify or complicate matters when much information needs to be sorted out.

**Threats to availability**	

a1	It usually takes more time to establish a phone call with video. Today this is usually a matter of a few seconds, time which may be saved in successful guidance trough video communication. The caller may however be negatively affected by delays during initiation of contact with the EMCC, which in turn may affect how the case is handled.
a2	The capacity of mobile phone networks is often reached during larger accidents. Videoconferencing demands more bandwidth than audio communication, which may be a problem when many people are calling at the same time. In some mobile networks video calls use a reserved bandwidth, not interfering with the bandwidth used for audio calls. Depending on traffic and network configuration, it can be easier or more difficult to make a call go through when using video calls.
a3, a4, a5	EMCCs commonly have audio logs of all communication with the public for playback. If the connection with the caller is lost and cannot be reestablished, audio playback may provide essential information to solve the emergency. Audio logs can also be useful for debriefing, or when questions later arise if the EMCC should have handled a case differently. If, for some reason, the log is not available, it may negatively affect patients in cases where connection is lost (threat a3). It may negatively influence the organization if logs are not available for documentation (threat a4) or debriefing (threat a5). There are several causes for these threats to occur, either that video is not recorded by default implementation, that playback of videorecordings is difficult, or that such recordings are corrupted or destroyed.
a6	Mobiles used for videoconferencing is kept out from the body and has greater exposure to weather conditions such as rain and cold temperatures. This may cause equipment failure and loss of connection.
a7	Technical difficulties because of less stable connection during mobile videoconferencing can delay or disrupt the exchange of information.
a8	Videoconferencing drain more battery on mobile phones than does audio communication. Use of video may therefore cause more lost connections. With empty batteries, communication can not be reestablished.
a9	In some situations the dispatcher may want to forward the call to another dispatcher either within the same EMCC or in a different EMCC. If this is not possible during video calls, the dispatcher may shut down the call and establish an audio call instead. This comes with a risk of wasting time.

**Threats to confidentiality**	

c1	Telephone communication can be wiretapped. While it takes more advanced technology and skills to wiretap a live videoconference over a mobile network, the public interest in images from emergencies suggest increased willingness to invest in such technology.
c2	Stored images are likely to be of greater interest and may contain more sensitive patient information than audio logs. Stored video and images may therefore increase attempts of unauthorized access.
c3	If visitors are allowed into the EMCC, or the images on computer screens can be observed by people outside the EMCC, this may reveal patient sensitive information. This threat is dependent on local conditions such as placement of computer screens and access restrictions to the EMCC.

**Threats to integrity**	

	No threats to data integrity were identified.

**Mixed threats**	

m1	With two-way videoconferencing the caller may identify the dispatcher. Dispatchers have been concerned that the loss of identity protection makes them more vulnerable to insults [7].
m2	The EMCC is a demanding work environment, and the introduction of videoconferencing may distract or increase demands on dispatchers. In the worst case, this may cause inefficiency or delays.
m3	The caller may focus on filming rather than helping the patient. The dispatcher may ask for images, and disturb or interrupt the treatment the caller otherwise would have initiated. Similar concerns were also raised when resuscitation instructions first was provided by telephone [29].

Different options for risk treatment were suggested. Several threats can be handled by proper implementation: the lack of availability of video logs (a3, a4, a5), the inability to forward video calls (a9), and unauthorized access to patient information (c2, c3). The loss of dispatchers' identity protection (m1) can be avoided with one-way videoconferencing, or by transmitting computer generated images of a dispatcher (an avatar) [18].

Some threats will be influenced by the intellectual capacity of dispatchers. Training of dispatchers may reduce the risk level of those threats, such as poor image quality (q2), misunderstandings due to interpretation of images or several patients in the same emergency (q3, q4, q5), and the images receiving too much attention from dispatchers or bystanders (m2, m3). For some dispatchers and in some situations the image may be helpful, while at times images can be an extra burden. EMCCs commonly use criteria based protocols for advice during emergencies [19-21]. Such protocols should be adapted for video based dispatch [7,14], which may contribute to reduction of the risk level for these threats.

The risk level of the remaining threats will be largely influenced by factors external to EMCCs, such as the sound quality (q1), time delays when establishing videoconferencing (a1), the capacity and security of the telecommunication networks (a2, a7, c1), weather conditions (a6), and the quality and capacity of callers' mobile phones (a7, a8). The risk level of these threats are likely to decrease with time, as technology and solutions mature. If users experience problems with sound quality or other technical problems during a video call, a switch to audio call may solve the problem, but with a loss of time.

## Discussion

This risk assessment identified twenty threats to information security for the use of mobile video calls between EMCCs and the public. None of these have a severe risk level (i.e., a combination of high consequence and likelihood). We have suggested ways to decrease or eliminate the risks, by proper implementation, organization, and staff training. Potential delays and poor sound quality were the greatest technical risks of mobile video calls. These threats are likely to decrease as technology improves.

Based on this risk assessment, we believe it is possible to implement videoconferencing from the public as a service in EMCCs with acceptable risks. However, some critical success factors of information systems in the organization will only be discovered during the implementation process [22]. A change in work environment may impose unacceptable loads on human cognitive abilities and potentially lead to errors, especially in a transition phase when new routines are being adopted [23]. When introducing a new service in the high stress environment of EMCCs, the process should therefore be closely monitored for unwanted incidents, even if unacceptable risks have not been identified at earlier stages. Risk assessment should be repeated at regular intervals to ensure that changes in environment, organization, or system do not introduce new unacceptable threats and that known threats do not increase in likelihood or consequence resulting in unacceptable risk levels for the system.

Risk assessment is a method for identification and evaluation of possible factors that may affect different aspects of change processes and their outcome, such as impact on services, organization, customers and users. Even the most thorough risk assessment process can miss out on some unforeseen consequences. ISO/IEC 27005:2008 outlines procedures for risk assessment, but several of the steps can be addressed by using different approaches. We used qualitative assessments by a multi-professional team. The composition of the team is important to cover different threats, but is no guarantee that all possible threats are found. Qualitative studies rarely give hard facts, but they can provide information and insight, and guide further research [24,25]. Our approach was prospective and addressed a future system at a high level, and has similarities with the Structured What-If Technique (SWIFT), which is a systematic team-oriented technique for hazard identification suitable for considering systems where human and organizational factors predominate [26,27]. Other methods for risk identification such as Hazard and Operability study (HAZOP), Failure Modes and Effects Analysis (FMEA), and Fault Tree Analysis (FTA) focus on process flow or hardware, and may be better suited for assessment of equipment details [26]. When risk assessments are carried out before new systems are implemented, sometimes even before they are constructed, it is not possible to do accurate measurements. Risk assessment as a scientific method therefore needs to be carried out in a systematic and critical fashion so that each issue can be discussed and debated openly. There is always a risk of bias in such discussions, resulting in overly positive or overly negative analysis. Our risk assessment was based on previous research in the field [7,9,14,28], and a part of systematic development of knowledge.

The result of risk assessments provides information for risk treatment (Figure 1), which involves decisions on how to reduce risk in an organization. The threats identified in this risk assessment should be used as input to formal requirements when planning and implementing video calls for EMCCs. The benefit of doing risk assessment before system implementation is that information security can be incorporated from the beginning.

For all health care service there are several risks involved - for the patients, for health care workers, for the organization, and for the service itself. Our risk assessment has only focused on the purpose of a communication system, namely information exchange and storage. Risks related to different types of patient conditions should be identified through clinical studies.

A threat may have different outcomes, from common incidents with no practical implications, to (very rarely) a chain of events with disastrous results. Poor sound quality, for instance, may be acceptable in many situations, but can in other cases cause misunderstandings that lead to worse patient treatment and possible patient death. For a new service there are no measurements of unwanted events, therefore assessments of associated consequence and likelihood become approximations. We found this led to a worst-case type of thinking that may have overestimated the risk level of some threats. Further studies are therefore needed to map type of errors and problems that may arise when videoconferencing is used during real emergencies.

## Conclusions

Video based communication with lay bystanders during prehospital emergencies may potentially improve the quality of prehospital patient care. In previous studies of simulated cardiac arrest, we have found that video calls are likely to improve confidence and reduce communication problems during prehospital medical emergencies [7,9]. In this risk assessment, we used qualitative methods to find potential threats to information security of using such video calls. This study has revealed several issues that should be considered carefully in requirement specifications for such systems. We did not identify potential threats with unacceptable high risk levels, which indicates that it is possible to implement the reception of video calls from the public in dispatch centers. The time is ripe to initiate a discussion on how emergency call centers should implement the new possibilites given by the mobile multi-media devices carried by a large portion of the population.

## Competing interests

The authors declare that they have no competing interests.

## Authors' contributions

SRB conceived of the study, and participated in its design and coordination, took part in the risk assessment group and drafted the manuscript. PH participated in the design of the study, took part in the risk assessment group and helped to draft the manuscript. EH participated in the design and coordination of the study, was leading the risk assessment and helped to draft the manuscript. All authors read and approved the final manuscript.

## Authors' information

SRB is an anesthesiologist (MD, PhD) with a background in computer science. PH is a computer scientist. EH is an expert on risk assessment with a background in computer science.

## Pre-publication history

The pre-publication history for this paper can be accessed here:

http://www.biomedcentral.com/1472-6963/11/244/prepub
